# Supplementary material for: Observation of an acoustic octupole topological insulator
Source: Nat Commun. 2020 May 15;11:2442. doi: 10.1038/s41467-020-16350-1 (PMC7229046; doi:10.1038/s41467-020-16350-1)
Supplement: Supplementary file 1 — Supplementary Information [file 41467_2020_16350_MOESM1_ESM.pdf]

Supplementary Information for

Observation of an acoustic octupole topological insulator

Xue et al.

Supplementary Note 1. Design of the acoustic octupole topological insulator.

To build an acoustic metamaterial that can be mapped to the desired tight-binding model, we use two types of building blocks: resonators and coupling waveguides. Each resonator is a hard-wall cavity filled with air, supporting modes at different frequencies. The mode of interest is a dipole mode with a nodal line (which can be used to achieve negative couplings). The cavity is designed to have a size of 80 mm × 40 mm × 10 mm, which maximises the separation between the dipole mode of interest and other modes (Supplementary Figure 1a). We couple the resonators with small coupling waveguides, which are also air-filled with hard walls. To realize couplings with different signs, we make the waveguide connect to different sides of the dipole nodal line (Fig. 1d in the main text). The coupling strength is controlled by the width of the waveguide.

The tight-binding model for an octupole topological insulator (TI) (Fig. 1b in the main text) is built from two quadrupole TIs with opposite coupling settings along the *z* direction. We first design the two layers as shown in Supplementary Figure 1b, c. These two layers are coupled along the third direction with dimerized coupling strengths, resulting in the acoustic octupole TI. Along the *z* direction, we use two coupling waveguides to connect adjacent resonators in order to ensure that the structure is mechanically stable.

With the preliminary design in hand, we proceed to optimize the system. There are several factors to consider. First, the lattice is anisotropic (for instance, the coupling strengths along *z* are not equal to those in the *xy* plane). Second, the coupling waveguides introduce resonance frequency shifts, which may be different at different sites. Third, the coupling waveguides do not only couple the dipole modes on different sites, but also other modes. Our simulation studies indicate that the first issue does not threaten to spoil the quantisation of the octupole moment, the other two might if their effects are strong enough.

To translate between the metamaterial and an equivalent tight-binding model, we use the Schrieffer-Wolff transformation method[1-4]. First, we solve the lowest 5 eigenmodes of a single resonator numerically (using COMSOL Multiphysics). Then we solve for the lowest 320 modes of a small lattice containing eight unit cells (64 sites, i.e. 5 modes per site, excluding modes introduced by coupling waveguides). We discretize the simulation data to produce matrices U_j, where the ith column contains the ith eigenmode at site j. This is projected onto the basis of single resonator eigenmodes by taking $P_j=(A^TA)^{-1}A^TU_j$, where the columns of matrix A contain the eigenmodes of a single resonator. Hence we obtain the coupling matrix $V=PDP^{-1}-H_0$, where $P=(P_1, P_2,\cdots, P_{63}, P_{64})^T$, D is a diagonal matrix containing the eigenfrequencies of the 8-unit-cell sample, and H_0 is a diagonal matrix containing the eigenfrequencies of the single resonator repeated 64 times. To restrict the effective description to the mode of interest, we perform the SW transformation perturbatively[2,4]. With the effective tight-binding model in hand, we tune the widths of the coupling waveguides and the distances between the resonators so that the nearest neighbour couplings along z are almost the same as the couplings in the xy plane, the resonance frequency shifts and long ranges couplings are small, and the frequency gap is large. Moreover, small holes are introduced to the resonators at the boundary sites, in order to shift their resonance frequencies to be the same as those in the bulk. In the final design, the lattice constants along x, y, z directions are 200 mm, 200 mm and 100 mm, respectively, and the widths for intra-cell (inter-cell) coupling waveguides are 3.2 mm (8 mm) in the xy plane and 2.68 mm (6.16 mm) along z direction. The final design has a ratio of $\gamma/\lambda \approx 0.18$ with $\gamma = 9.8\times(1\pm4\%)$ Hz and $\lambda = 53.3\times(1\pm4\%)$ Hz, and the resonance frequencies of the eight resonators in the unit cell are $f_0 = 2145.1\times(1\pm0.05\%)$ Hz. The dispersion relation calculated from the effective tight-binding model matches full-wave simulation results (Supplementary Figure 1d). Due to the large separation between the target mode

and other modes, the next-nearest couplings in the extracted tight-binding model are quite small (Supplementary Figure 1e, f). Thus, the designed acoustic lattice deviates almost negligibly from the ideal tight-binding model.

Supplementary Note 2. Nested Wilson loops and topological invariants.

From the effective tight-binding model obtained by the method described in previous section, we calculate the topological numbers from the nested Wilson loop method[5,6]. First, we calculate a Wilson loop along *z* direction for the four bands below the bandgap, yielding 2D Wannier bands (Supplementary Figure 2a). This splits the original four bands, which are almost degenerate in frequency, into two Wannier sectors (labelled v_z^+ and v_z^- in Supplementary Figure 2b) that are spatially separated along *z*. Being gapped, the two Wannier sectors carry their own topological invariants. Next, we calculate a nested Wilson loop within one of the Wannier sectors (here we choose v_z^-) along *y*, which again yields two separated Wannier bands, denoted v_y^+ and v_y^- in Supplementary Figure 2c. One more nested Wilson loop over one of the sectors (again we choose v_y^-) gives the Wannier sector polarization along *x* as p_x^v=0.49. Similar procedures can be done for all directions (the nesting order of the Wilson loops makes negligible difference) to obtain all three polarizations $\{p_x^v, p_y^v, p_z^v\}$=$\{0.49, 0.48, 0.49\}$. This result indicates that the couplings between the mode of our interest and other modes, and the effects of resonance frequency shifts, are negligible.

Supplementary Note 3. Verification of π flux.

Although the most remarkable feature of an octupole TI is the topological corner states located at corners of a finite sample, corner states can also appear in systems without an octupole moment[7-9]. Thus, it is important to check the bulk topology to ensure the corner states come from a bulk

octupole moment. Here we verify the π flux in the limit of λ→0 (Supplementary Figure 3b) and γ→0 (Supplementary Figure 3c) to ensure our design maps to the tight-binding lattice shown in Fig. 1b. As shown in Supplementary Figure 3d, f, the measured spectra when the source and microphone are placed at the same site (R1 or R7) features two peaks, corresponding to two branches of eigenmodes (denoted by black circle on the horizontal axis). When we fix the source at R1 (or R7) and measure the acoustic field over all eight sites, features of π flux on each facet of the cubic are observed[10,11]. The intensity on the resonators located at diagonal positions of the excitation is almost zero, and the phases on the resonators adjacent to the excitation are 0 (π) if the coupling is positive (negative) (Supplementary Figure 3e, g).

Supplementary Note 4. Profile and stability of corner states.

Similar to the Su–Schrieffer–Heeger model[12], the corner states are highly concentrated at the corner and decay exponentially into the bulk with a decay length of $2/\log(\gamma/\lambda)$[1]. To test this experimentally, we measure the decay of the corner states along x, y and z hinges and compare with the exponential curve given by the ratio $\gamma/\lambda \approx 0.18$. As shown in Supplementary Figure 4a-c, the measured results match well with the theoretical prediction.

As can be seen in Supplementary Figure 4a-c, the corner states are sublattice polarized. This gives a unique stability to the corner states under some local perturbations. Here two types of perturbations are considered (see inset of Supplementary Figure 4f): resonance frequency shifts on the three sites next to the corner (perturbation 1) and a resonance frequency shift on the corner site (perturbation 2). For perturbation 1, we tune the resonant frequencies of the three sites next to one of the corners to be $f_0(1+df_i)$, where f_0 is the unperturbed resonant frequency and df_i are random numbers uniformly distributed from -δ to δ. Supplementary Figure 4d shows calculated

eigenfrequencies with different perturbation strength δ. As can be seen, the corner state is almost unaffected since the corner state has neglectable distribution on the perturbed sites. For perturbation 2, the perturbation is added on one of the corner sites. This perturbation leads to a frequency shift on one of the corner states (Supplementary Figure 4e). To test above results experimentally, we deliberately introduce metal balls into the resonators, which shifts the resonance frequency of a single resonator about 10 Hz. As shown in Supplementary Figure 4f, the spectra measured at the corner show that frequency of the corner state stay unchanged for perturbation 1 and changes for perturbation 2, which are consistent with numerical calculations. To further confirm the peaks in Supplementary Figure 4f correspond to corner states, we mapped out the field distributions at the peak frequencies and found the sound is indeed localized around the corner (Supplementary Figure 4g-i). This unique stability of corner states under local perturbations may be useful for further applications in sensing devices.

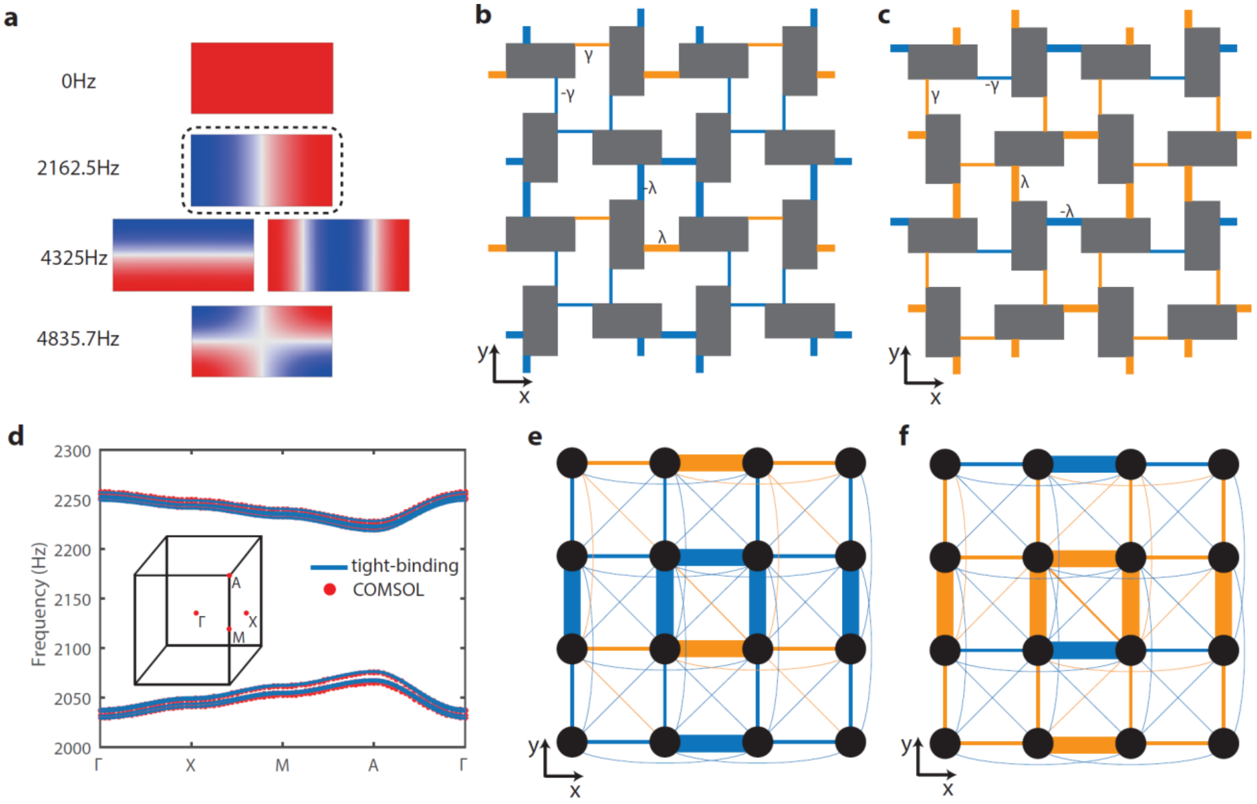

Supplementary Figure 1 Design of the acoustic octupole topological insulator. a Lowest five eigenmodes of an isolated acoustic resonator with size 80 mm × 40 mm × 10 mm. The dashed box denotes the mode of interest. **b, c** Two layers of the designed acoustic lattice. Grey rectangles represent resonators and orange (blue) ones denotes coupling waveguides for positive (negative) couplings. **d** Bulk dispersions of the designed acoustic lattice along high symmetry lines calculated from the effective tight-binding model (blue lines) and full-wave simulations (red dots). **e, f** Illustrations of the effective tight-binding model extracted from simulations. **e** and **f** show the in-plane configurations for the bottom and top layers, respectively. The dots denote the sites, and the width and color of the lines correspond to the strength and sign of the couplings, respectively. The nearest neighbour coupling along *z* have the same strength as those in the *xy* plane, and the next-nearest couplings along *z* are negligible.

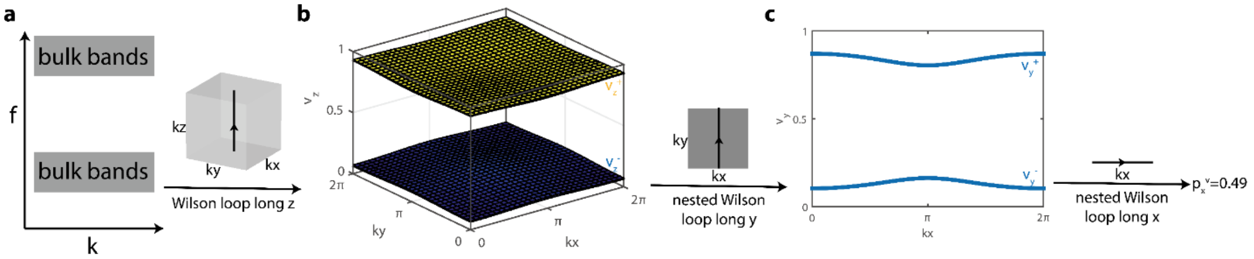

Supplementary Figure 2 Nested Wilson loops and Wannier bands. a Illustration of a Wilson loop along *z* for the four bands below the bandgap. **b** The resulting 2D Wannier bands (left) which are used to calculate the nested Wilson loop along *y* (right). **c** The resulting 1D Wannier bands whose sectors carry nontrivial polarizations along *x*.

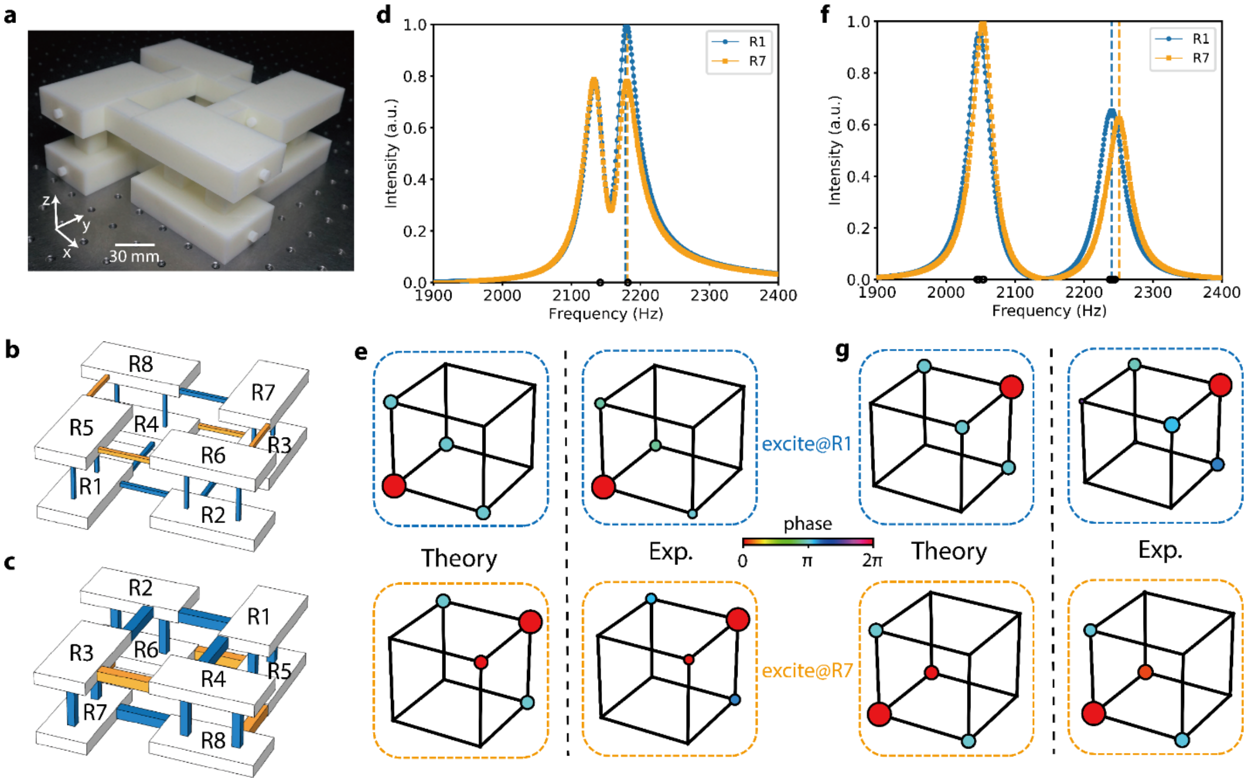

Supplementary Figure 3 Identification of π flux in the limits of vanishing inter-cell and intra-cell couplings. a Photo of a sample with vanishing intra-cell couplings ($\gamma = 0$). **b** Schematic of the isolated cubic sample in $\lambda \rightarrow 0$ limit. **c** Schematic of the isolated cubic sample in the $\gamma \rightarrow 0$ limit. **d** Measured spectra of the structure shown in **b**. The results for excitation and detection at R1 (R7) are plotted in blue (orange). Black circles on the horizontal axis indicate simulated eigenfrequencies. **e** Left panel: theoretically calculated eigenmodes. Right panel: experimentally measured field distributions at peak frequencies (denoted by vertical dashed lines in **d**) when source is placed at R1 (blue dashed box) and R7 (orange dashed box). **f, g** Similar to **d, e** but for the structure shown in **c**. In **e-g**, the radius of the balls indicates amplitude and color denotes phase.

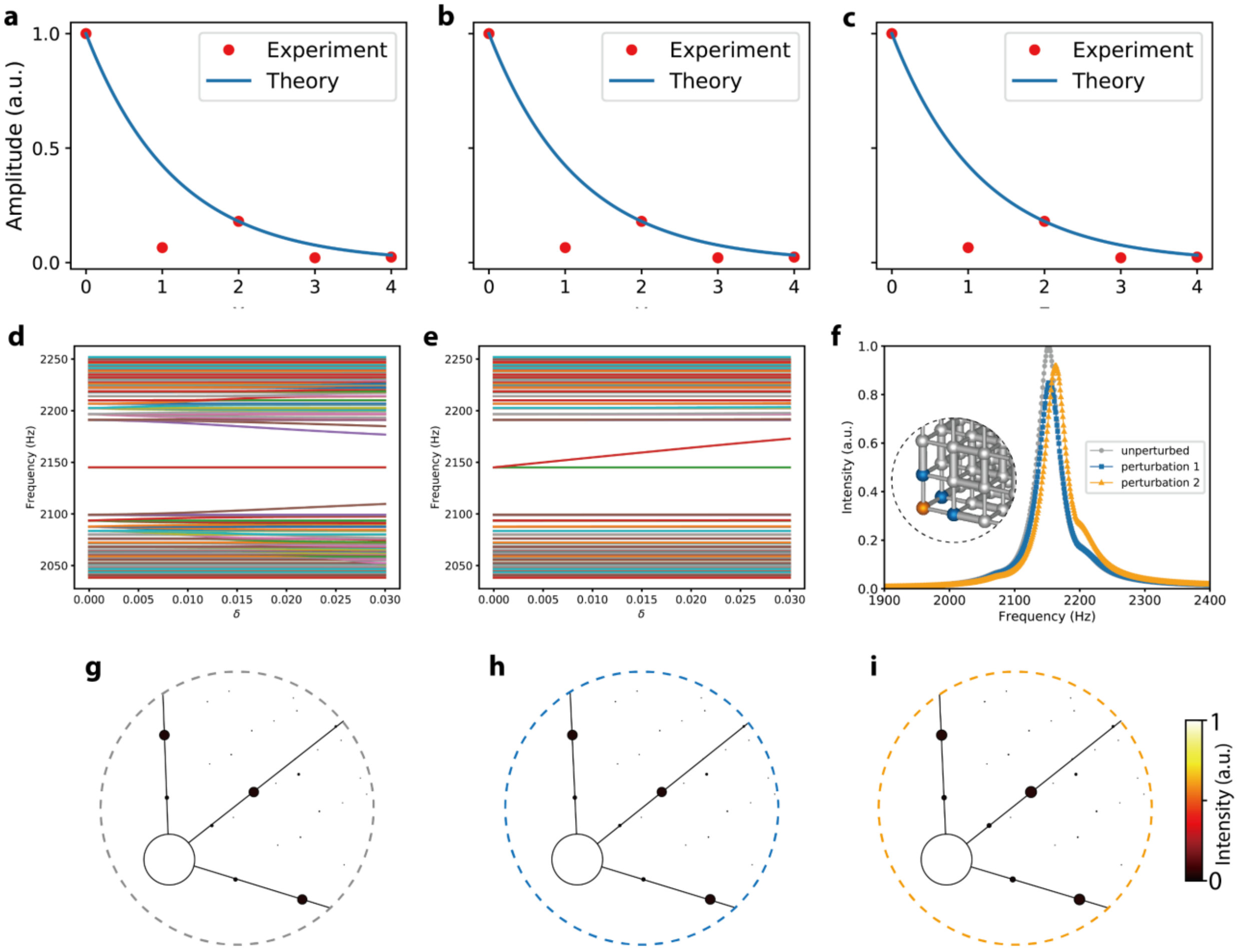

Supplementary Figure 4 Profile and stability of corner states. a Measured acoustic pressure (red dots) along x-directional hinge. The measured results agree well with the exponential decay curve (blue line) predicted from the ratio γ/λ. Here the horizontal axis refers to the site index along x-directional hinge where site "0" is the corner site. **b**, **c** the same as **a** but for y (**b**) and z (**c**) directions. **d**, Eigenfrequencies of a finite lattice versus on-site perturbation strength δ, where the perturbation is applied to the resonance frequencies of the three sites adjacent to one of the corner sites. **e**, Eigenfrequencies of a finite lattice versus perturbation strength δ, where the perturbation is applied to the resonance frequency of one of the corner sites. In the calculations, we took a lattice consisting of five unit cells along each direction with f_0 = 2145.1 Hz, γ = 9.8 Hz and λ = 53.3 Hz. **f**, Experimentally measured spectra at the corner site for different cases suggested in the inset. **g-**

i, Experimentally measured field distributions for the three cases. In these measurements, the speaker is fixed at the corner site and the microphone scans over all sites. Signal outside the plotting range is neglectable and thus is not shown.

Supplementary References

1. Serra-Garcia, M. *et al.* Observation of a phononic quadrupole topological insulator. *Nature* **555**, 342–345 (2018).

2. Matlack, K. H., Serra-Garcia, M., Palermo, A., Huber, S. D. & Daraio, C. Designing perturbative metamaterials from discrete models. *Nat. Mater.* **17**, 323-328 (2018).

3. J. R. Schrieffer, P. A. Wolff, Relation between the Anderson and Kondo Hamiltonians. *Phys. Rev.* **149**, 491–492 (1966).

4. S. Bravyi, D. P. DiVincenzo, D. Loss, Schrieffer–Wolff transformation for quantum many-body systems. *Ann. Phys.* **326**, 2793–2826 (2011).

5. Benalcazar, W. A., Bernevig, B. A. & Hughes, T. L. Quantised electric multipole insulators. *Science* **357**, 61-66 (2017).

6. Benalcazar, W. A., Bernevig, B. A. & Hughes, T. L. Electric multipole moments, topological multipole moment pumping, and chiral hinge states in crystalline insulators. *Phys. Rev. B* **96**, 245115 (2017).

7. Xue, H. et al. Realization of an acoustic third-order topological insulator. *Phys. Rev. Lett.* **122**, 244301 (2019).

8. Weiner, M., Ni, X., Li, M., Alù, A. & Khanikaev, A. B. Demonstration of a third-order hierarchy of topological states in a three-dimensional acoustic metamaterial. *Sci. Adv.* **6**, eaay4166 (2020).

9. Zhang, X. et al. Dimensional hierarchy of higher-order topology in three-dimensional sonic crystals. *Nat. Commun.* **10**, 5331 (2019).

10. Peterson, C. W., Benalcazar, W. A., Hughes, T. L. & Bahl, G. A quantized microwave quadrupole insulator with topologically protected corner states. *Nature* **555**, 346–350 (2018).

11. Keil, R. et al. Universal sign control of coupling in tight-binding lattices. *Phys. Rev. Lett.* **116**, 213901 (2016).

12. Su, W. P., Schrieffer, J. R. & Heeger, A. J. Solitons in polyacetylene. *Phys. Rev. Lett*. **42**, 1698–1701 (1979).
